# Navigating the cultural geography of indigenous peoples’ attitude toward genetic research: the Ohana (family) heart project

**DOI:** 10.3402/ijch.v72i0.21346

**Published:** 2013-08-05

**Authors:** May Vawer, Patsy Kaina, Ann Leonard, Michael Ogata, Beth Blackburn, Malia Young, Todd B. Seto

**Affiliations:** 1 Center for Outcomes Research and Evaluation, The Queen's Medical Center, Honolulu, HI, USA; 2 Hana, Maui, HI, USA; 3 Departments of Medicine and Native Hawaiian Health, John A. Burns School of Medicine, University of Hawaii, Honolulu, HI, USA

**Keywords:** Native Hawaiian, qualitative, heart failure, biological specimens, interviews, screening

## Abstract

**Background:**

Little is known about the burden of heart failure among indigenous populations, including Native Hawaiians (NH). Recent concerns about genetic research in the NH community resonate with similar concerns raised by American Indian, Alaskan Native and Canadian First Nations communities and have raised questions about the best way to proceed with studies involving biological specimens.

**Objective:**

To help us plan a study to investigate disparities in heart failure incidence and outcomes in a NH community, we performed a qualitative study to examine the community's expectations for heart failure research that includes the collection of biological specimens.

**Methods:**

Eighty-five NH with a personal or family history of heart failure, who lived in a geographically isolated community in the state of Hawai‘i participated in 1 of 16 semi-structured interviews. Interviews were conducted in a standard manner, with open-ended questions designed to explore their expectations for a heart failure research study that included the collection of biological specimens. Interviews were analyzed thematically through iterative readings and coding.

**Results:**

Four key themes regarding heart failure research with the use of biological specimens characterized their expectations: (a) Need to foster trust between investigator and community; (b) Establish a partnership with the community to identify needs and goals; (c) Need for mutual benefit to investigator and community; (d) Identification of a key voice to represent the community. Participants expressed strong support for research. However, the strength of that support was directly related to the strength of the relationship between the research team and the community. The collection of biological specimens for genetic analyses was not an explicit concern or barrier per se.

**Conclusions:**

It appears feasible to conduct a heart failure research study that includes the collection of biological samples. However, success will likely require addressing the community's expectations, including the need for a long-term partnership built on trust and mutual benefit, and a key voice to represent the community.

Heart failure (HF) is a major cause of death and disability that affects more than 5 million people in the US, and over 20 million worldwide (1,2). The burden of HF varies across racial and ethnic groups, with African-Americans and Hispanics suffering poorer quality of life, more frequent hospitalizations and decreased survival compared to Caucasians (3). Less is known about the burden of HF among indigenous populations. American Indians and Alaskan Natives (AI/AN) suffer from HF more often than the general US population (4), although their age-adjusted death rates are lower than the rates for Blacks and Caucasians (5). Indigenous Australians, including Aboriginal and Torres Strait Islander peoples, have a HF prevalence and mortality rate that is more than double the rate among Non-Indigenous Australians (6,7). Like Indigenous Australians, Native Hawaiians (NH) appear to develop HF at a younger age, with recent data suggesting that HF among NH is also more often non-ischemic and associated with more severe left ventricular dilatation and systolic dysfunction (8).

Research involving biological specimens among NH and other indigenous populations has occasionally faltered. The historical role of US institutions as colonizers, definers of identity and appropriators of culture has led to mistrust (9–11), as have traditional academic research practices, in which investigators, rather than communities, define the research agenda, methods, and interpretation and dissemination of study results (10–14). Thus, concerns over a project to map the NH genome (13) and a study of a novel genetic disorder in a NH family (15) reverberated in the NH community in a manner that echoes similar concerns raised by AI/AN (16) and Canadian First Nations (17) communities.

For the past 4 years, our group has worked closely with a predominantly NH community, which has a high prevalence of HF. Our initial pilot work focused on interviewing family members and developing pedigree charts to better understand the burden of HF in the community. Based on these interviews, our findings suggest a high rate of familial HF, with an autosomal dominant pattern of inheritance, and a high prevalence of cardiovascular disease risk factors, including diabetes, hypertension and obesity. Encouraged by colleagues to obtain biological specimens to detect potentially novel genetic variants associated with cardiomyopathy, we were also aware of concerns raised by the NH community about genetic research (13,15). For example, a proposal by the University of Hawaii in 2003 to patent the NH genome was strongly opposed by the NH community, which viewed it as another unwelcome imposition of a Western concept (property ownership) upon cultural beliefs that also countered NH perspectives on the dynamism and shared ownership of traditional knowledge (13). The strength of these concerns resulted in the termination of the study and a resolution by the Association of the Hawaiian Civic Clubs “urging the University of Hawaii to cease development of the Hawaiian Genome Project or other patenting or licensing of Native Hawaiian genetic material until such time as the Native Hawaiian people have been consulted and given their full, prior and informed consent to such project” (18).

It is for these reasons that we chose to follow the path of investigators, who worked with AI/AN communities to gain an understanding of their perspectives on research (19–22). The goal of this study was to work with our community partners to assess their expectations for a HF research study that includes the collection of biological specimens, with the intent that the findings from this qualitative exploration will guide the development and implementation of a future HF study.

## Methods

### Study population

We used a purposive sampling strategy to enroll individuals with a personal or family history of HF who lived in a small (population ~1,200), geographically isolated community with a large NH population (~40%) in the state of Hawai’i. Participants in our initial study, in which we interviewed individuals with a personal or family history of HF, in order to develop pedigree charts and assess the burden of HF in the community, were eligible to enroll. Individuals were invited to participate by invitation letters and direct contact by our community-based investigator (PK). Adults at least 18 years old and self-identified as being NH were included, with a specific effort to include young adults and kupuna (elders) to provide a broader perspective. This study was performed as part of the University of Hawaii's Center for Native and Pacific Health Disparities Research (NIH-NIMHD P20 MD000173) and received approval from The Queen's Medical Center Institutional Review Committee.

### Data collection

We conducted 16 semi-structured, in-depth interviews during 2 extended visits to the community in 2010–11. Given norms of respect for elders in the NH community, and recognition that younger participants may be reluctant to speak freely in front of their elders, participants were grouped broadly by age and chose to be interviewed one-on-one, or in groups of 2 or more. At the suggestion of our community partners, all participants underwent a limited health screening (vital signs, brief physical exam) upon enrolment.

Interviews were moderated by a single investigator. Two assistants acted in a supportive role, audio-recorded the interviews, took notes and assisted with transcription. All interviews were conducted in English and were 60–120 min in duration. Each session started with a brief introduction, including an overview of HF, HF screening and genetic testing. Interviews were conducted in a standardized manner, using an interview topic guide with open-ended questions designed on the basis of clinical experience and discussion among the study team. Questions were selected to understand participants’ views on a HF research study that includes biological specimens. Examples of questions are included in Table I.

**Table I:** Example interview questions

What comes to mind when you think of genetic testing?
What about genetic testing for research?
What are some questions you might have?
What are your thoughts about participating in a heart failure research study that will examine your genes?
What would make you interested in participating?
What would make you not want to participate?

### Analysis

Transcripts of the semi-structured interviews were analyzed through a series of iterative readings. A single investigator reviewed each transcript and identified and coded the emergent domains and key concepts. For validity and reliability, 2 co-investigators independently read the transcripts and reviewed the identified coding and domains that emerged from the data. Disagreements were resolved by jointly recoding the relevant portions of the transcript, and through discussion and reaching consensus among the investigators. Credibility (believability of results), authenticity (adequate representation of multiple realities of those being tested), criticality (critical self-reflection) and integrity (adequate checks on interpretation) were assessed throughout the data collection and analysis process to optimize study validity (23).

## Results

Eighty-five individuals agreed to participate in the study and provided written informed consent. By design, all participants were NH and had either a personal or family history of HF. Participants ranged in age from 18 to 90 years, with over 60% female (Table II). A substantial proportion of participants were part of a large, multigenerational extended family, with an extensive history of HF.

**Table II:** Participant description (n =85)

Age, mean±SD	52±19.0 years
Native Hawaiian	100%
Female	63.5%
BMI, mean±SD	34.9±10.6
History of HF	37.6%
Family history of HF	97.6%

There was broad agreement that the community was in distress, with a heavy burden of heart disease, diabetes and cancer. “Too much make (death),” as 2 participants noted, particularly among the younger and middle-aged adults. With that recognition, there was a strong interest in participating in research studies that might improve the community's health. Four broad key themes characterized participants’ expectations for a HF research study that includes biological specimens. All themes were expressed by participants across the age groups (Table III).

### Need to foster trust between investigator and community

A strongly identified theme was the need to foster trust between investigator and community. There was a deep suspicion of investigators who might “just grab the information and run and disappear … (and) don't come back.” The need to develop an on-going relationship, with history and depth, was clearly stated, with the recognition that a meaningful relationship would lead to greater research opportunities. Two paths to develop this relationship were identified. The first, transparency of intent and action, addressed concerns about hidden agendas (“they don't share their goals with the community”) and being unwitting research subjects. It was also recognized that the transparency should be in both directions, with the community articulating their expectations to the research team and, in turn, the research being receptive to that feedback. The second, time and commitment, reflects the long-term perspective held by the participants, who generally looked beyond the next study, and saw a continuum of projects and studies derived from the on-going partnership. Trust is developed through a long-term relationship and a continued presence in the community by the research team through regular communication and site visits. Ideally, the research team becomes part of the community's family. One participant states, “it's all about the relationship and it's all about the love …. It's a slow process.”

**Table III:** Four key themes

Establish partnership with community to identify needs and goals
Need to foster trust between investigator and community
Need for mutual benefit to investigator and community
Identification of a key voice to represent the community

With a trusting relationship, participants were more willing to participate in research studies, including those that collected biological specimens for genetic analyses. Having food available for study subjects was considered a priority for many participants, reflecting its important role in cultural and family events. A participant explained, “we need research so we can have hope, and maybe help our kids and grandkids who have the heart problems in the family.”

### Establish partnership with community to identify needs and goals

Participants readily identified examples of prior research studies that were brought into the community by “outside” investigators over the years. It was felt that these investigators were often more interested in the community's weaknesses than its resources and strengths, which created a sense of powerlessness and exploitation. One participant noted that other investigators “have come for all the diseases – diabetes, cancer, heart – and it's all about what they want … we all know that. So when they come, like that, we stay home.” Another noted that “no researcher asked what we wanted or needed … to help with our health.” Participants wanted community resources and strengths acknowledged and, when possible, utilized. Cultural practices, including hula and traditional kanaka maoli lapa'au (Hawaiian medicine), were specifically mentioned as a key resource and strength.

Participants clearly expressed their expectation that researchers partner with the community in the development and implementation of the HF study. Although participants did not state the need to direct or co-direct the study, they did suggest that, when possible, community members should help to run the study (e.g. recruitment, administrative tasks, data collection). One participant suggested that the community take a more active role in defining the overall goals: researchers “can figure out the reasons why (they suffer from HF) and then we embark on the solutions, and then it's up to the community to take the next steps.”

### Need for mutual benefit to investigator and community

There was a strong belief that research studies should provide tangible benefit for the community as well as the investigators. “Too many people from outside come in, grab data, and never see again.” Thus, a study that involves the collection of biological specimens for genetic analyses may be more acceptable if the study provides a clear benefit to the community and the participants’ families. Even if there was no direct benefit to the participants, they were willing to participate if the rationale for the study was clearly described and if there was potential benefit to their family or community in the future. That benefit may take different forms. For example, education about HF and its risk factors, and programs that promote physical activity, particularly cultural activities such as fishing and hula, were identified as significant needs, and a potential way to more broadly affect the community, including the younger generations. Participants felt strongly that investigators provide education about HF, including how it can “run in the family” and how it can be treated. “We need education, we need help for connecting the dots, our community needs to know about their heart health, and how they can change it …. My mother's generation was illiterate about their health, about seeing the doctor, about their disease, they didn't understand … they didn't even know what questions to ask.”

The concept of a HF research study that included biological specimens was supported if it would allow both investigators and participants to benefit. The research team could identify subjects with HF and possible familial cardiomyopathy that may be eligible for research studies. Participants supported screening tests that provided immediate feedback and could be readily understood. The plan to include screening echocardiography, which shows left ventricular function in real-time, was widely supported, as were blood tests for diabetes (glucose, HbA1c), and vital signs. Education on diet and exercise was widely supported. Several participants felt that the information from the screening tests should be shared with their primary care physicians.

### Identification of a key voice to represent the community

Participants stated the importance of identifying a community member to serve as a liaison with the research team and represent the community as a key voice. One participant notes that he “wouldn't come if had outsiders … need to have a connection in the community who can speak to researchers and who we trust.” This individual should be a respected leader in the community, and be able to help the research team with communication, study design and implementation, and address issues/concerns as they arise. Conversely, it was also felt that the research team should have an individual that serves as a liaison with the community, and that this person should be knowledgeable about the community, and trusted by the community and the key voice. It may or may not be the primary investigator of the study.

## Discussion

In this study, we interviewed NH with a personal or family history of HF to understand their expectations for a HF research study that includes biological specimens for research. Our findings were both reassuring and enlightening. Participants expressed strong support for research and a willingness to enroll as study subjects. However, the strength of that support was directly related to the strength of the relationship between the research team and the community. The collection of biological specimens for genetic analyses was not an explicit concern or barrier per se, but its support would be unlikely if the investigator did not have a long-term, meaningful partnership with the community that was built on trust. We could not identify a shortcut or abbreviated path for the investigator to travel.

The impetus for our study was based on our need to move forward, beyond the development of pedigree charts demonstrating a high prevalence of heritable HF. Navigating that path forward, however, was complicated by recent events that raised concerns by members of both the scientific and NH community about the viability of genetic research. In one instance, a NH family in a geographically isolated community was asked to submit blood samples, which led to the discovery of a rare genetic disease. The family was generally unaware of the investigator's plans or findings, was not asked to provide written informed consent, and affected family members were left without provisions for genetic counselling or treatment (15). Soon thereafter, an effort to obtain biological samples to patent the NH genome was viewed by the NH community as an unwelcome imposition of Western concepts upon traditional culture. (13) Similarly, plans to genetically modify and patent 3 lines of taro, the traditional staple of the Hawaiian diet that is culturally identified as an ancestor of the NH people, met with significant resistance from the NH community (24). These issues echoed similar concerns raised by AI/AN (16) and Canadian First Nations (17) communities, and by projects such as the Human Genome Diversity Project and the Genographic project, in which researchers have sought to collect genetic samples from indigenous communities for reasons unrelated to community benefit (25).

Our results support the community-based participatory research (CBPR) approach used by investigators who have worked with indigenous people, including NH (26) and AI/AN populations (19,20–22,27, –29). Three of the four themes identified from our participants’ interviews fit explicitly within the CBPR paradigm (establish partnership, foster trust, need for mutual benefit), with the fourth (identify key voice to represent the community) fitting implicitly, as a means to develop and maintain a relationship based on trust and open communication (27). Our study also supports the recommendations for researchers proposed by Hiratsuka and colleagues, who performed a qualitative study to examine Alaska Native people's perceptions, understandings and expectations for research involving biological specimens. Although the goal of their study differed from ours, their recommendations address 2 themes that we identified in our study: (a) establish a partnership with the community to identify needs and goals; and (b) foster trust, including transparency of intent and action (20).

Although participants identified the importance of a liaison to represent the community, they did not articulate the need for a community member to serve as a leader or co-leader on the research team. However, participants did express a desire to participate in operational aspects of the study (e.g. recruitment, data collection), with more interest in taking a leadership role in a follow-up studies, once investigators better understood the causes of HF in the community (“… it's up to the community to take the next steps.”). This suggests that, in this NH community, the need to involve community members as study leaders or co-leaders, a standard practice for CBPR studies, may depend on the type of study, and should be openly discussed early in the planning process. Indeed, in our more recent work with this community evaluating a culturally tailored obesity intervention, community members expressed a strong desire to actively lead the development and implementation of the study, and coordinate the dissemination of the study results.

There are several limitations to our study. First, we interviewed participants from a small, geographically isolated, rural community, and our results may not be generalizable to other communities. Similarly, our participants had a personal or family history of HF, and our findings may not generalize to other conditions. Second, the intent of this qualitative study was to understand the expectations for a HF research study that includes biological specimens. It was not intended to provide a detailed understanding of the community's perceptions and expectations of genetic research, or specific information on the collection and handling of biological specimens. Further qualitative studies, similar to recent work with AI/AN populations (20,29), are needed to obtain a fuller and more nuanced understanding of the issues surrounding genetic research in this NH community.

In summary, it appears feasible to conduct a HF research study that includes biological specimens in this NH community. However, success will likely require addressing the community's expectations, including the need for a long-term partnership built on trust and mutual benefit, and a key voice to represent the community. A culturally informed relationship grounded in a CBPR approach is a key factor in identifying and overcoming potential barriers and designing a successful study.

## References

[1] American Heart Association (2007). Heart Disease and Stroke Statistics — 2007 Update.

[2] Joseph SM, Cedars AM, Ewald GA, Geltman EM, Mann DL (2009). Acute decompensated heart failure: contemporary medical management. Tex Heart Inst J.

[3] Kaholokula JK, Saito E, Mau MK, Latimer, Seto TB (2008). Pacific Islanders’ perspectives on heart failure management. Patient Educ Couns.

[4] Center for Rural Health (2010). Native aging facts: congestive heart failure among older American Indians and Alaska Natives. http://ruralhealth.und.edu/pdf/fs_heartfailure.pdf.

[5] US National Institutes of Health: National Heart, Lung, and Blood Institute (2012). Morbidity & mortality: 2012 chart book on cardiovascular, lung, and blood disease.

[6] AIHW: Penm E (2008). Cardiovascular disease and its associated risk factors in Aboriginal and Torres Strait Islander peoples 2004–05.

[7] McGrady M, Krum H, Carrington MJ, Stewart S, Zeitz C, Lee GA (2012). Heart failure, ventricular dysfunction and risk factor prevalence in Australian Aboriginal peoples: the Heart of the Heart Study. Heart.

[8] Mau MK, Asao K, Efird J, Saito E, Ratner R, Hafi M (2009). Risk factors associated with methamphetamine use and heart failure among native Hawaiians and other Pacific Island peoples. Vasc Health Risk Manag.

[9] Bowekaty MB (2002). Perspectives on research in American Indian communities. Jurimetrics.

[10] Walters KL, Simoni JM (2009). Decolonizing strategies for mentoring American Indians and Alaska Natives in HIV and mental health research. Am J Public Health.

[11] Manson SM (1989). The Barrow Alcohol Study: emphasis on its ethical and procedural aspects. Am Indian Alsk Native Ment Health Res.

[12] Cook BP (2001). A call for respect and equality for indigenous scholarship in Hawaiian health. Pac Health Dialog.

[13] Singeo L (2007). The patentability of the Native Hawaiian genome. Am J Law Med.

[14] Weiss KM, Richard H (2003). Ward, Ph.D. (June 7, 1943–February 14, 2003): wild ride of the Valkyries. Am J Hum Genet.

[15] Chang RM, Lowenthal PH (2001). Genetic research and the vulnerability of Native Hawaiians. Pac Health Dialog.

[16] Andrews LB, Buenger N, Bridge J, Rosenow L, Stoney D, Gaenssien RE (2004). Ethics. Constructing ethical guidelines for biohistory. Science.

[17] Wiwchar D (2000). Bad blood. Ha-Shilth-Sa.

[18] Association of Hawaiian Civic Clubs (2003). Resolution urging the University of Hawai'i to cease development of the Hawaiian genome project or other patenting or licensing of Native Hawaiian genetic material until such time as the Native Hawaiian people have been consulted and given their full, prior and informed consent to such project. http://www.ipcb.org/pdf_files/hi_GenReso.pdf.

[19] Williams RL, Wilging CE, Quntero G, Kalishman S, Sussman AL, Freeman WL (2010). Ethics of health research in communities: perspectives from the southwestern United States. Ann Fam Med.

[20] Hiratsuka VY, Brown JK, Hoeft TJ, Dillard DA (2012). Alaska Native people's perceptions, understandings, and expectations for research involving biological specimens. Int J Circumpolar Health.

[21] Buchwald D, Mendoza-Jenkins V, Croy C, McGough H, Bezdek M, Spicer P (2006). Attitudes of urban American Indians and Alaska Natives regarding participation in research. J Gen Intern Med.

[22] Christopher S, Watts V, McCormick AKHG, Young S (2008). Building and maintaining trust in a community-based participatory research partnership. Am J Public Health.

[23] Whittemore R, Chase SK, Mandle CL (2001). Validity in qualitative research. Qual Health Res.

[24] Santos L (2008). Genetic research in native communities. Prog Community Health Partnersh.

[25] MacIntosh C (2005). Indigenous self-determination and research on human genetic material: a consideration of the relevance of debates on patents and informed consent, and the political demands on researchers. Health Law J.

[26] Nacapoy AH, Kaholokula JK, West MR, Dillard AY, Leake A, Kekauoha BP (2008). Partnerships to address obesity disparities in Hawai'i: the PILI 'Ohana Project. Hawaii Med J.

[27] Sahota P (2010). Community-based participatory research in American Indian and Alaskan Native communities.

[28] Christopher S, Saha R, Lachapelle P, Jennings D, Colclough Y, Cooper Y (2011). Applying indigenous community-based participatory research principles to partnership development in health disparities research. Fam Community Health.

[29] Boyer BB, Mohatt GV, Pasker RL, Drew EM, McGlone KK (2007). Sharing results from complex disease genetics studies: a community based participatory research approach. Int J Circumpolar Health.

